# Extremophilic Microorganisms as a Source of Emerging Enzymes for the Food Industry: A Review

**DOI:** 10.1002/fsn3.4540

**Published:** 2024-12-30

**Authors:** Tolulope Joshua Ashaolu, Tanu Malik, Rakhi Soni, Miguel A. Prieto, Seid Mahdi Jafari

**Affiliations:** ^1^ Institute for Global Health Innovations Duy Tan University Da Nang Vietnam; ^2^ Faculty of Medicine Duy Tan University Da Nang Vietnam; ^3^ Centre of Food Science and Technology CCS Haryana Agricultural University Hisar Haryana India; ^4^ Department of Microbiology CCS Haryana Agricultural University Hisar Haryana India; ^5^ Nutrition and Bromatology Group, Department of Analytical Chemistry and Food Science, Faculty of Science Universidade de Vigo Ourense Spain; ^6^ Department of Food Materials and Process Design Engineering Gorgan University of Agricultural Sciences and Natural Resources Gorgan Iran; ^7^ Halal Research Center of IRI, Iran Food and Drug Administration, Ministry of Health and Medical Education Tehran Iran

**Keywords:** emerging enzymes, extremophilic microorganisms, extremozymes, food industry

## Abstract

Modern‐day consumers are interested in highly nutritious and safe foods with corresponding organoleptic qualities. Such foods are increasingly subjected to various processing techniques which include the use of enzymes. These enzymes like amylases, lipases, proteases, xylanases, laccases, pullulanase, chitinases, pectinases, esterases, isomerases, and dehydrogenases could be derived from extremophilic organisms such as thermophiles, psychrophiles, acidophiles, alkaliphiles, and halophiles. As these organisms can grow under severe environmental conditions, they can produce functional enzymes (extremozymes) used in producing safe foods (such as gluten‐free, lactose‐free, lower acrylamide, or lower trans‐fat products). The extremozymes also enhance nutrient bioavailability and bioaccessibility (e.g., predigested nourishments like baby formulae), and improve nourishment functionalities such as surface, sensory, and bioactive properties. Therefore, exploring alternative sources of enzymes for better compatibility and long‐term adaptability in the processing stages is a promising approach for obtaining novel food products. This review will establish novel discovery methods of extremozymes from psychrophiles, thermophiles, acidophiles, alkaliphiles, and halophiles, the enzymes' types, mechanisms of action, and their food applications. It will also contribute to their commercial relevance and the furtherance of their discovery.

## Graphical abstract

Enzymes such as amylases, lipases, proteases, xylanases, laccases, pullulanase, chitinases, pectinases, esterases, isomerases, and dehydrogenases could be derived from extremophilic organisms like thermophiles, psychrophiles, acidophiles, alkaliphiles, and halophiles. These enzymes or extremozymes are applicable in the production of safe foods (such as gluten‐free, lactose‐free, lower acrylamide, or lower trans‐fat products), enhancing nutrient bioavailability and bioaccessibility (e.g., predigested nourishments like baby formulae), and improving nourishment functionalities such as surface, sensory, and bioactive properties.

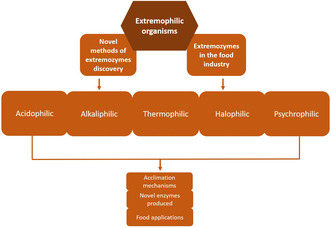

## 1 Introduction

Extremophiles are microorganisms that thrive under intense temperatures, for example, those above the boiling point or below the freezing point of water. Furthermore, certain bizarre species are able to survive in acidic or alkaline environments under heavy pressure, or in an environment having high saltiness. Extremophiles are mainly classified as thermophiles (e.g., *Anaerobranca gottschalkii*), hyperthermophiles (e.g., *Pyrococcus furiosus*), and psychrophiles (e.g., *Lacinutrix algicola*) (Schröder et al. 2020). The most famous classes among them are psychrophiles and thermophiles. The thermophiles can be obligate or facultative, thriving at very high temperatures that could reach up to 80°C while the psychrophiles (also known as cryophiles) can grow and reproduce in temperatures as low as −20°C, as they are usually found in permanently cold places such as the deep sea or polar regions of the earth. Extremophiles are described in each of the three areas of life, for example, archaea, prokaryotes, and eukaryotes. For example, psychrophilic microbes are signified by bacteria, archaea, algae, or yeasts but also by plants and animals (Buzzini et al. 2012). The first extremophile *Thermus aquaticus* was found in the Yellowstone park in the western United States (Koonin and Martin 2005). Further studies have isolated extremophiles from diverse extreme environments, such as deep sea, acid mine drainage, deserts, and cold regions (Javaux 2006; Daletos et al. 2017; Ibrar et al. 2020).

To survive in these environments, extremophiles have developed different strategies to cope with extreme conditions and the lack of nutrients; among them, the production of extremozymes (EXZs), enzymes that catalyze chemical reactions under rough conditions. Therefore, extremophiles are ideal organisms to investigate adaptative strategies to increase stress tolerance. Research on genetics and structural data related to extremophiles and EXZs could provide useful data for the design of new bioprocesses in food, pharmaceutical, and biotechnological industries, due to their high versatility, and also for bioengineering of nontolerant enzymes currently employed in those industries. In fact, EXZs came into the limelight some decades ago but continuous technological advancements in genetics and enzymology are expanding the potentials of EXZs activity and use in multifarious industries. To date, several companies have applied enzymological engineering from psychrophiles and thermophiles to attain target properties usable on commercial scales of operations (Hermann et al. 2018). The usual conditioning of common enzymes cannot meet certain industrial demands, for instance, the ability to withstand sinusoidal temperature and pH differences, warranting the need for EXZs. These novel enzymes are multibillion‐dollar in value and are expected to keep growing at more than 4% rate per year (Zhu et al. 2020).

In this review, therefore, we focused on psychrophilic, thermophilic, acidophilic, alkaliphilic, and halophilic EXZs. Novel discovery methods of EXZs, the enzymes' types, mechanisms of action, and their food applications were discussed. The food applications of carbohydrases such as amylases, β‐galactosidases, and pectinases were mentioned while discussing their commercial relevance. Furthermore, glucoamylases, proteases, and laccases which are of great commercial significance as heat‐tolerating enzymes were also critically surveyed.

## 2 Novel Discovery Methods of Extremozymes: An Overview

The most common method of discovering enzymes of all types of origins is the conventional culture‐dependent whereby the microorganisms of interest are grown in cell cultures, and characterized for their enzyme production capabilities, and then resulting in screening out the target enzymes. The challenges surrounding this traditional strategy are many but chief among them with regard to this present review is that the psychrophilic and thermophilic microorganisms that only grow and replicate in extreme environmental conditions with huge commercialization prospects cannot be grown this way. They thrive best in extremely cold or hot conditions, and discovering them requires more than just the conventional discovery mechanisms. Novel techniques recently gained the public spectrum include metagenomics, to capture microorganisms that cannot be readily isolated or grown. The concept is to effectively discover new or characteristic genes and enzymes which have bypassed culture‐based methods. To this end, uncultivatable EXZs such as extremophilic lipases and esterases from the deep blue sea extremophilic organisms have been screened for their metagenomes (López‐López, Cerdan, and Gonzalez Siso 2014; Madhavan et al. 2017; Jin et al. 2019).

More often than not, the use of the metagenomics approach will involve certain screening in culture plates to ascertain the activity of the enzyme in question. In order to ascertain an enzyme's activity, for instance amylase implies the use of a starch‐iodine staining test (Jin et al. 2019). Metagenomics can be classified as sequence‐based or function‐based, both of which involve the genomic DNA isolation from the environment directly (Datta et al. 2020). The highly sought‐after enzymes are identified due to their conserved sequences or else due to their expressed features/activity in both sequence‐based and function‐based approaches, respectively. A common technique used in the sequence‐based approach is hybridization. It is used to screen metagenomic clones with the aid of an oligonucleotide primer or probes to capture the genes of interest. The target genes are then amplified with the aid of a polymerase chain reaction (PCR) thermocycler non‐specific or specific primers to be expressed in suitable vectors. The genes of interest may be obtained other than this method. All that must be done is to do a retrieval of their sequences from metagenomics data directly immediately after bioinformatic elucidations. The genes are then submitted into de novo synthesis and codon optimization (Popovic et al. 2015). As for the function‐based approach, the genes are identified based on their activities or functions and therefore do not require the sequence information. However, the expression of genes in this method may be affected by misconfiguration or misfolds, inefficient translation, poor recognition, and defective post‐translational modification of proteins. Several broad‐host vectors enabling various expression levels have been used to resolve these challenges, making function‐based strategy the preferred one for EXZ screening (Jin et al. 2019).

The sequence‐based approach can help with novel sequence discovery which could be compared with already known existing sequences, an efficient means of EXZ discovery. The main problem with this method is its dependence on bioinformatic elucidation, connoting that several novel sequences or functional enzymes and genes may be bypassed. On the other hand, the function‐based approach solely identifies genes based on their functions and not sequences. This helps to overcome analytical errors such as arriving at the same gene sequences with conflicting functions. Once discovered, these EXZs play crucial roles in the food industry. Based on various factors like temperature, pH, salt concentration, pressure, and water accessibility, extremophiles that are responsible for the production of EXZs are placed among a broad range of categories like alkaliphiles, acidophiles, thermophiles, psychrophiles, barophiles, xerophiles, radiophiles, and halophiles (Elleuche et al. 2014), as summarized in Table 1.

**TABLE 1:** Description of extremophiles, their habitats, and extremozymes produced.

Extremophile category	Habitat	Examples	Extremozymes
Thermophiles	Hot springs, hydrothermal vents, acidic geothermal spring, solfataric fields	*Bacillus subtilis* NS 8, *Bacillus* sp., *Geobacillus* sp., *Thermus aquaticus*, *Pyrococcus woesei*	Lipase, proteases, gelatinase, amylase, glucoamylase, xylanase, pullulanase
Psychrophiles	Antarctic lake water, arctic marine sediment, deep cold Pacific Ocean waters, Chinese Yellow Sea	*Candida antarctica*, *Clostridium* sp., *Flavobacterium*YS‐80, *Anoxybacillus* species	Lipase, protease, amylase
Acidophiles	Acidic geothermal spring, solfataric fields, hot springs, coal and copper mines	*Bacillus* species, *Sulfolobussol fataricus*, *Acidiphilium angustum*, *Metallspaeraprunae*	Amylases, proteases, glucoamylases, cellulases, endonuclease
Alkaliphiles	Hydrothermal vents, marine sediments, hot spring	*Bacillus* sp. MLA64, *Bacillus subtilis* DR8806, *Geobacillus* sp., *Saccharopolyspora*s sp., *Anoxybacillus* sp.	Lipase, proteases, gelatinase, amylase, glucoamylopullunase
Halophiles	Hydrothermal vents, salt lakes	*Bacillus* sp., *Geobacillus* sp., archaeal strains	Lipase, protease, esterase, amylase
Piezophiles	Hydrothermal vents	*Pyrococcus* sp.	DNA polymerase
Radiophiles	Antarctic valley, wastewater treatment Plant	*Deinococcus* sp., *Radiodurans* sp.	Lipase
Microaerophiles	Hot springs	*Sulfurihydrogenibium azorense*	Azoreductase

*Source:* Kochhar et al. (2022), Swaminaathan et al. (2024), Wang et al. (2024).

## 3 Current and Prospective Roles of Extremozymes in the Food Industry

Enzymes are responsible for speeding up the pace at which biological substances are broken, collected, and transformed from one form to another. These molecules are a fascinating “green” biocatalyst class. They are non‐toxic and safe for the environment (since they are biodegradable), can operate under mild circumstances, have brilliant substrate specificity, and are suitable for catalysis of a wide range of processes (Akanbi, Agyei, and Saari 2019). In the food industry, EXZs aid in the production of safe foods (such as gluten‐free, lactose‐free, lower acrylamide, or lower trans‐fat products), aid in the improvement of nutrient bioavailability and bioaccessibility (as in predigested nourishments like baby formulae), and play a key role in enhancing nourishment functionalities such as surface, sensory, and bioactive properties. Extremophiles are an excellent source of highly active and stable extremophilic enzymes, such as amylases, lipases, proteases, xylanases, pullulanase, chitinases, pectinases, esterases, isomerases, and dehydrogenases (Akanbi, Agyei, and Saari 2019). Food and beverage, animal feed, detergents, biofuels, leather, pulp and paper, and textiles are just a few of the industries where EXZs have become important tools. Additional advancements in business sectors such as diagnostics, pharmaceuticals, research, and development give them top priority because they are directly related to human health diagnostics and treatment.

**FIGURE 1:**
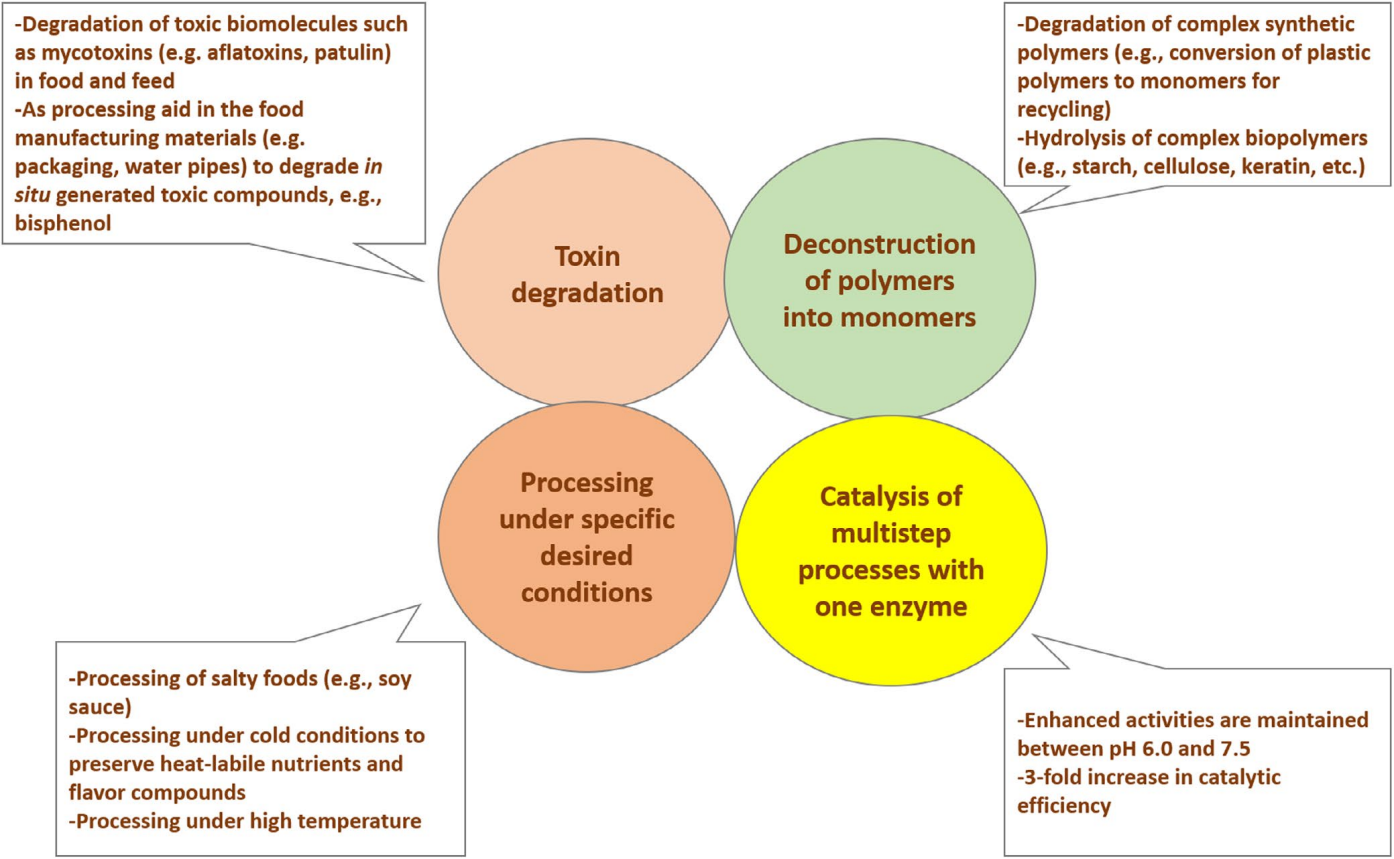
Role of extremozymes in the food industry.

EXZs from psychrophilic, thermophilic, acidophilic, alkaliphilic, and halophilic microorganisms play a central role in the food industry like (i) toxin degradation; (ii) deconstruction of polymers into monomers; (iii) breakdown of multistep processes with one enzyme; and (iv) processing under specified desired conditions such as cold, heat, acidic, alkaline, and saline (Figure 1). As a result, all of the above points compel us to conduct a study of the available literature, with a focus on the role of psychrophilic and thermophilic EXZs in food processing and preservation. It also comes to our knowledge that this sector has been undisturbed until now and has been studied in such a critical manner that it opens up new vistas for researchers in a variety of fields, including food, biotechnology, and pharmaceuticals.

### 3.1 Toxin Degradation

EXZs may have an advantage in degrading hazardous chemicals formed in situ as a result of food processing conditions such as heat treatments. This family of chemicals includes nitrosamines, heterocyclic aromatic amines, chloro‐propanols, furans, trans fatty acids, and acrylamide. Furthermore, since heat is required for the creation of these chemicals, heat‐stable enzymes can be used to limit or stop their creation. The use of heat‐stable CGTase for instance is crucial with regard to toxic and organic waste reduction during the production of cyclodextrins from starch (Horikoshi 1999). However, there is no proof of the use of extremophiles in the breakdown of any of the aforementioned in situ generated hazardous chemicals. This is therefore, a knowledge gap and a prospective role that warrants comprehensive investigation.

### 3.2 Deconstruction of Polymers Into Monomers

EXZs could also be used to prospectively break down complex biopolymers into monomer building blocks. Enzymatic deconstruction processes improve the treatment or valorization of polymeric by‐products, the treatment of biotechnological materials, and the production of high‐value hydrolysates from complex polymers (Bell et al. 2022; Corrêa et al. 2022). In wine production for example, using cell wall deconstructing enzymes makes the reduction of tannin‐polysaccharide interactions possible leading to the availability of more tannins in the final wine (Osete‐Alcaraz et al. 2020).

### 3.3 Breakdown of Multistep Processes

In multistep food processing businesses, EXZs that perform multiple biocatalytic activities have been used. Keratinases are an example since they are enzymes that break down keratinous proteins and can be found in hair, horns, wool, feathers, hooves, and nails (Vidmar and Vodovnik 2018; Luan et al. 2019). Recently, *Geobacillus thermoleovorans* NP33 thermostable amylopullulanases have been identified to be capable of one‐step liquefaction‐saccharification, converting starch into sugars in a single step (Akanbi, Ji, and Agyei 2020). The enzymes were thermostable (half‐life of 7.8 h at 90°C) and capable of digesting raw starch without the need for Ca^2+^ as a cofactor.

### 3.4 Processing Under Specific Desired Conditions Such as Cold, Heat, Acidic, Alkaline, and Saline

In this context, several examples can be mentioned: ß‐galactosidase is used to remove lactose from chilled milk; pectinase is used to reduce viscosity and turbidity in chilled juice; amylase is used to hydrolyze polysaccharides in the starch manufacturing industry, and cold‐active proteases are used to treat meat. The great molecular stability of cold‐active enzymes has led to their novel uses (Van den Burg 2003). More about these extreme conditions will be discussed in the sections below. Table 2 is a summary of some EXZs and their applications in the food industry.

**TABLE 2:** Summary of the applications of extremozymes in the food industry.

Industry	Extremozyme	Purpose
Dairy	Rennet (protease)	Coagulant in cheese production
	Lactase	Hydrolysis of lactose to give lactose‐free milk products
	Protease	Hydrolysis of whey proteins
	Catalases	Removal of hydrogen peroxide
Alcohol production	Ayloglucosidase	Conversion of starch to sugar
Brewing	Cellulases, β‐glucanases, α‐amylases, proteases, maltogenic amylases	For liquefaction, clarification, and supplement malt enzymes
Baking	α‐amylases	Breakdown of starch, maltose production
	Protease	Breakdown of proteins
	Glucose oxidase	Stability of dough
Fruit juice	Pectinase	Increase of yield and juice clarification
	Glucose oxidase	Oxygen removal
Meat	Protease, trypsin, aminopeptidases	Meat tenderizing
	Papain	Breakdown of various components

*Source:* Bankar et al. (2022), Kochhar et al. (2022), Ashcroft and Munoz‐Munoz (2024), Rawat, Chauhan, and Pandey (2024).

## 4 Psychrophilic Microorganisms and Their Enzymes

Psychrophiles (cryophiles) can efficiently grow at a low temperature ranging from −20°C to 10°C. More than 75% of our planet is located in regions that experience extraordinarily low temperatures (≤ 15°C). The cryophiles are cold‐loving microorganisms generally found from the Arctic to the Antarctic regions, deep‐sea, snow‐covered land, and glaciers of the land surface (Sathiyanarayanan et al. 2017). For example, *Planococcus halocryophilus* Or1, isolated from high Arctic permafrost, grows and replicates at temperatures as low as −15°C (Mykytczuk et al. 2013). Other cold conditions where cryophiles are found include cold‐water lakes, cold soils, cold deserts, and caves. They can flourish and maintain their metabolic cycles in these omnipresent regions (Dhaulaniya et al. 2019).

### 4.1 Acclimation Mechanisms of Psychrophilic Microorganisms

Low compound activity and low enzymatic rates, modified medium systems, and protein cold‐denaturation are only a few of the challenges that psychrophiles confront when living in cold conditions (D'Amico et al. 2006). These bacteria require diverse characteristics to live in extreme environments, ranging from particular cell structures to stable catalysts for executing certain metabolic activities efficiently (Chung et al. 2020). For example, psychrophiles produce greater amounts of unsaturated and methyl‐extended unsaturated fats, as well as restricted acyl chain length unsaturated fats, to prevent the loss of membrane fluidity (Chintalapati, Kiran, and Shivaji 2004). They also produce cold shock proteins and chaperones to aid their protein folding, protect RNA and protein synthesis, and maintain the fluidity of cell membranes, among other functions (Phadtare 2004). Essentially, all components of cells, including cold‐adjusted catalysts that are adaptable and maintain clear‐cut activities at low temperatures, should be acclimated to cold conditions.

As the mean kinetic energy available for responses is insufficient at low temperatures to overcome the energy barrier of protein initiation, enzymatic activity is reduced. When temperatures drop, proteins denature due to a reduction in water molecule accessibility as molecules become less linked with proteins (Karan, Capes, and DasSarma 2012). Cold‐adjusted (or psychrophilic) substances have a variety of particular adjustments in their underlying structure, making them more flexible than mesophilic and thermophilic proteins. This characteristic explains why enzymatic activity is high at low temperatures (Siddiqui and Cavicchioli 2006). To maintain their metabolic activities and thrive in cold conditions, psychrophiles have created remarkable adaptive procedures at the quality and protein levels. Adaptive strategies include changes in transcription and translation, membrane fluidity, expression of the cold‐shock protein, production of antifreeze/ice‐nucleating proteins, compatible solutes, exo‐polysaccharides, and biocatalysts capable of catalyzing biochemical reactions at low temperatures, to mention some adaptative strategies (Baraúna et al. 2017; Koh et al. 2017).

### 4.2 Food Applications of Psychrophilic Extremozymes

Psychrophilic EXZs can be classified based on their potent utilization: (i) food additives EXZs, (ii) food ingredients EXZs, and (iii) preparing aids EXZs. Generally, the use of EXZs as food additives can help to preserve the flavor of food and enhance the taste and appearance of the product. For example, stabilizing and preservative activity is shown by invertase and lysozyme enzymes (a group of EXZs), which are approved by the European Commission (EC) as food additives. In the dairy industry, EXZs are required for the preparation of dairy products. For example, lactase derived from *Kluveromyces lactis* has been used to make lactose‐free goods; since this enzyme hydrolyzes the lactose present in milk (Mateo et al. 2004). In addition, EXZs are used as food additives in the beverage and bakery industries to effect and maintain both color and clarity, respectively.

For reasons such as preventing contamination and spoilage, maintaining labile and unpredictable flavor mixes, minimizing unwanted chemical reactions that can occur at higher temperatures, and having a greater level of control over cold‐active enzymes, which can be inactivated at high temperatures, low‐temperature processes are now given more attention in the food and beverage industry than high‐temperature processes (Pulicherla et al. 2011). Low‐temperature processes reduce the risk of mesophile contamination while also saving energy (Marx et al. 2007). As a result, a slew of cold‐adjusted enzymes for food and beverage applications have been identified and produced. Different psychrophilic enzymes for food applications are covered in the following sections.

#### 4.2.1 Amylases

Amylases are one of the most important microbial hydrolytic enzymes produced by normal and stress‐adaptive bacteria for all starch‐related industries (Yadav 2021). Most amylases used in the food and beverage sector are thermophilic at the moment, although this is changing due to the benefits of adopting cold‐active enzymes. The most cold‐active α‐amylase investigated came from the Antarctic bacteria *Alteromonas haloplanktis* (*Pseudomonas haloplanktis*), and it was effectively transferred to mesophilic bacteria such as *E*. *coli* (Médigue et al. 2005). In addition, a further study reported that this enzyme also presents a significant halotolerance between NaCl concentrations 0.01 and 4.5 M, compared to mesophilic and thermostable α‐amylases (Srimathi et al. 2007). These results suggested that psychrophilic adaptation also conferred halotolerance, which increases its versatility for industrial applications. Other promising psychrophilic α‐amylase was isolated from *Aeromonas veronii* NS07, which was reported to be active in a wide range of temperatures (0°C–50°C), with optimum temperature at 10°C (Samie et al. 2012). Recently, a study optimized the production of α‐amylase from psychrophilic and halophilic *Shewanella* sp. ISTPL2. The enzyme remained active in a broad range of temperatures (4°C–70°C) and pH (5–10). Specifically, the highest activity of the extracellular enzyme was 10,064 U/mL after 12 h of incubation at 10°C and pH 6.9 (Rathour et al. 2020). Other study characterized a novel cold‐active glucoamylase from *Saccharophagus degradans*. After expression in *E*. *coli*, authors reported a high stability at low temperatures, although optimum activity was observed at 39°C, with a high specificity towards maltose (Wayllace et al. 2021). These enzymes, along with other cold‐active amylases like the extracellular amylase recovered from *Microbacterium foliorum* GA2 (Kuddus et al. 2012) and the cold‐active amylase obtained from the marine bacterium *Z*. *profunda* (Qin, Huang, and Liu 2014), are gaining popularity in the food and beverage industry for applications, such as beer and wine fermentation, bread production, and fruit juice production.

#### 4.2.2 β‐Galactosidases

These enzymes play a fundamental role in dairy industry, where they are employed to obtain lactose‐free products (Nivetha and Mohanasrinivasan 2017), by hydrolyzing lactose into glucose and galactose (Mahoney 1998; Wheatley et al. 2013). Mesophilic microorganisms with temperature optimums in the 30°C range produce many industrial galactosidases (Zolnere and Ciprovica 2017). Incorporating cold‐active galactosidases into this reaction could improve process stability while cutting costs. Improved cold‐active galactosidases are a must‐have for particular enterprises due to their numerous applications. Several microorganisms have been studied in this regard, including *Arthrobacter* sp., *Pseudoalteromonas* sp., *Paracoccus* sp. or *Halomonas* sp., among other species (Wang et al. 2020). For example, the production of cold‐active β‐galactosidade from *T*. *frigidphilosprofundus* has been optimized, reaching an optimum of 10,657 U/mL, which was much higher than the results obtained with *Pseudoalteromonas haloplanktis*, another well‐known psychrophilic microorganism (Pulicherla et al. 2013). Recently, four β‐galactosidase genes from psychrophilic *Cryobacterium* sp. LW097 were successfully transferred to *E*. *coli* and were characterized in detail. The enzymes showed different substrate preferences and optimum temperatures, but they presented a high activity at 5°C. The hydrolytic activity of the enzymes was investigated in cold milk, showing that it can be affected by the concentration of the substrate and some cations. Despite this, the high substrate specificity of one of the enzymes makes it interesting for further research in low‐temperature lactose hydrolysis (Wang et al. 2020). In this field, some patents have been developed. For example, Stougaard and Schmidt were granted a patent for a cold‐active galactosidase, obtained from genus *Alkalilactibacillus* sp. with stable enzymatic activity at temperatures below 8°C. The patent is intended for the production of low‐lactose dairy products and can also be applied in some pharmaceuticals (Stougaard and Schmidt 2012).

#### 4.2.3 Pectinases

Pectinase enzymes are divided into three categories: polygalacturonase (PG), pectinesterase (PE), and pectin lyase (PL). To break down the pectin polymer into smaller bits, these enzymes use hydrolysis, trans‐elimination, and de‐esterification. These enzymes are extensively used in the fruit and vegetable processing, wine, coffee, and olive oil extraction industries, among others (Kashyap et al. 2001). For example, pectinases are often used to clarify juice to reduce turbidity, viscosity, and astringency, as well as to enhance color and yield (Kumar and Mondal 2015). Nowadays, pectinases generated from mesophilic living organisms are the most cost‐effective. However, the practice of cooking meals at low‐temperatures is opening the way for new chilly‐adjusted pectinases. Filamentous fungus such as *Aspergillus* sp., bacteria such as *Bacillus* sp., and yeasts such as *Aureobasidium pullulans* produce this cold‐active pectinases (Reddy and Sreeramulu 2012; Aaisha and Barate 2016) and several studies have reported their potential in the food industry. For example, cold‐active pectinases of several strains of *A*. *pullulans*, isolated from grapes, maintained their activity under different concentrations of glucose, ethanol, and SO_2_, commonly found during vinification. These results suggest their possible application in low‐temperature winemaking (Merín and de Ambrosini 2015). A study investigated a cold‐active pectinase from the psychrophilic yeast, *Cystofilobasidium capitatum* strain PPY‐1, reporting a high activity at 10°C, although it was not the optimum temperature. Parameters *K*_m_ and *V*_max_ for pectin were 36.6 mg/mL and 3000 units/mg, correspondingly, showing that this enzyme effectively degrade pectin at a low temperature (Nakagawa et al. 2005). More recently, the production of cold‐active enzymes, including pectinases, was investigated in several yeast isolates. Specifically, maximum pectinase activities were reported for *Yamadazyma* (fungal) isolates, which could be considered for further optimization studies and industrial applications, including baking, cheese production, and meat tenderization (Daskaya‐Dikmen, Karbancioglu‐Guler, and Ozcelik 2018).

## 5 Thermophilic Microorganisms and Their Enzymes

Thermophilic microorganisms are those that can survive at high temperatures. Thermophiles and hyperthermophiles, respectively, dwell in settings that are hotter than 50°C–80°C. For instance, *Pyrolobus fumarii*, a chemolithoautotrophic archaea, has been demonstrated to live at 113°C, whereas *Methanopyrus kandleri*, a methanogenic hyperthermophile, has been shown to exist at 122°C (Blöchl et al. 1997; Cowan 2004). Thermophiles and hyperthermophiles thrive in hot springs and deep‐sea vents, where temperatures often exceed 100°C, such as geysers and volcanic zones (Takai et al. 2008). Sulfate‐dependent cells are among the most significant thermophiles and hyperthermophiles. Sulfate elimination was discovered in the hyperthermophilic archaea, *Archaeoglobus fulgidus*, as one of the oldest energy processes (Beeder et al. 1994). In species that flourish in these adverse environments, sulfate oxidation is frequent (Kletzin et al. 2004). The oxidation of organic molecules and metals, which is impacted by low pHs, has also been demonstrated.

Only a few of the thermophilic enzymes that have been researched include cellulases, amylases, pullulanases, xylanases, mannanases, pectinases, chitinases, proteases, lipases, esterases, and phytases (Sunna and Bergquist 2003). The enzymes of thermophilic bacteria are capable of proteolysis in acute conditions such as the presence of denaturing agents, organic solvents, and high salinity. The advantages of using these enzymes include the ability to minimize contamination risk, maintain low adhesiveness, and increase substrate solubility (Raddadi et al. 2015).

### 5.1 Acclimation Mechanisms of Thermophilic Microorganisms

Proteins from hyperthermophiles have sparked a lot of attention since thermostable enzymes have become more significant in biotechnological operations. Comparative investigations of mesophilic and thermophilic enzymes and other proteins have revealed that, in the majority of cases, amino acid sequences are closely conserved, despite some compositional variations. The thermophiles has characteristic physical properties as well as electrostatic interactions that enable them to remain active at high temperatures. Thermophiles have a variety of adaptations, such as the ability to maintain their shape and function under high temperatures. The formation of disulfide liaisons between two ions with opposite charges by thermophiles will increase the number of hydrophobic deposits (Mayer et al. 2012). Glutamate dehydrogenase from *P*. *furiosus* develops at 105°C and has a higher concentration of hydrophobic amino acids and a lower concentration of polar and charged amino acids than mesophilic forms of the enzyme. Furthermore, the number of glycine residues was reduced (Maras et al. 1994). When examining how thermodynamic factors affect protein stability, these findings are not surprising. The protein exerts a stabilizing effect due to dense packing structures with tight hydrophobic cores that limit solvent contact with the nucleus. A decline in protein surface area as a function of protein size, which has been identified in *P*. *furiosus* proteins, is another aspect that contributes to increased thermostability. The hydrophobic cores of *P*. *furiosus* enzymes are tightly packed, with numerous ionic pairs and submerged atoms, which tend to limit unfavorable solvent interactions (Kumar and Nussinov 2001).

### 5.2 Food Applications of Thermophilic Extremozymes

Temperatures between 80°C and 110°C and pH = 4.0–7.5 are ideal for hyperthermophilic enzymes, while 50°C–80°C are suitable for thermophilic enzymes in which they perform at their optimum.

#### 5.2.1 Glucoamylases

Glucoamylase, also known as amyloglucosidase or amylase, is an exoenzyme that degrades starch into its constituents, namely—d‐glucose. These enzymes can survive a wide pH range and are particularly thermostable (Zheng et al. 2010). In the food industry, glucoamylases are used to break down dextrin into basic sugars, manufacture high‐glucose and high‐fructose syrups, sake, soy sauce, and light beer. They are also utilized in the baking industry to improve the color of bread crusts and the quality of high‐fiber baked goods by increasing flour consistency, reducing dough staling, and improving the color of bread crusts and the quality of high‐fiber baked goods (Blanco et al. 2014; James, Simpson, and Marshall 2009). These enzymes have been isolated from different extremophiles including *Bacillus acidocaldarius*, *Thermoanaerobacter tengcongensis*, and *Picrophilus torridus*. For example, a study characterized a glucoamylase‐type enzyme obtained from *B*. *acidocaldarius* RP1 isolate, showed an optimum stability at 65°C and pH = 4.5, with a *V*_max_ of 600 mU/mg and *K*_m_ of 11.7 mg/mL (Natalia et al. 2002). Other authors studied a glucoamylase from *T*. *tengcongensis* after expression in *E*. *coli*. In this case, the enzyme displayed the highest activity at 75°C and pH 5 and was specifically active towards maltose (80 U/mg) (Zheng et al. 2010). Thermophilic Archaea *Thermoplasma acidophilum*, *Picrophilus torridus* and *P*. *oshimae* have been reported to produce glucoamylases highly stable in extreme thermal and acidic conditions. Maximum activity was reported at 90°C and pH 2 (Serour and Antranikian 2002). Several species of thermophilic fungi have been investigated. For example, a glucoamylase from *Rhizomucor pusillus* was expressed in *Pichia pastoris* and was further characterized. The results showed high thermostability, with maximum activity (1237 U/mL) at 70°C and pH = 4.0 (He et al. 2014). More recently, a novel thermostable glucoamylase has been obtained from thermophilic *Thermoanaerobacter ethanolicus*. This enzyme was successfully expressed in *E*. *coli*. Further characterization revealed a high thermal and pH stability at 75°C and pH = 5 as the optimum values. The enzyme showed a high activity (75.3 U/mg) against maltose, but it also was able to hydrolyze soluble starches from potato, rice, and corn. In addition, moderate content of ethanol did not significantly affect its stability (Wayllace et al. 2022), suggesting that this enzyme may be a potential candidate for starch processing.

#### 5.2.2 Proteases

Hydrolases that break down peptide links in proteins to produce hydrolysates, peptides, or amino acids are known as proteolytic enzymes (EC: 3.4). Dietary enzymes called proteases are the second most common type of proteolytic enzymes (Akanbi, Agyei, and Saari 2019). The majority of commercial enzyme formulations have traditionally been developed for the laundry industry, although they play a fundamental role in the food industry. Meat tenderization, beer/wine clarity, and the production of dairy products like cheese are only some of the applications of proteases. These enzymes are considered highly stable, that is, temperature, pH, salt, and organic solvents do not affect the majority of proteolytic EXZs found in the literature (Sarmiento, Peralta, and Blamey 2015). Proteases such as papain, rennin, laccases, subtilisin, thermitase, proteinase K, lantibiotic peptidase, kexin, pyrolysin, and proteolysin are produced by a range of extremophilic Archaea and bacteria (Shinde and Thomas 2011). For instance, Aqualysin I, a proteinase K subgroup extracellular protease obtained from the thermophile T. *aquaticus* YT‐1 has demonstrated a significant heat stability. This enzyme was found to be stable across a broad pH range (5–13) and to have maximum activity at 70°C after being expressed in *P*. *pastoris* hosts (Olȩdzka, Da̧browski, and Kur 2003). Another study reported that *Halobacterium* sp. strain HP25 produced a protease significantly resistant to a wide range of conditions of salinity, acidity, and temperature (Elbanna, Ibrahim, and Revol‐Junelles 2015). It was determined that optimum conditions were 17% NaCl, pH 8, and 60°C, with a specific activity of 6,350 U/mg. These characteristics make this enzyme a potential candidate for several applications. In the food industry, it could be used in the ripening of salted fish, for instance (Elbanna, Ibrahim, and Revol‐Junelles 2015).

Other authors produced a protease from *Thermoascus aurantiacus* by solid‐state fermentation. Optimum values of enzyme activity were reported at 60°C and pH 5.5. Also, the proteolytic activity was assessed on skim milk, showing an elevated hydrolysis of caseins (Merheb et al. 2007). A similar study demonstrated the production of rennet from the thermophilic fungus, *Thermomucor indicae‐seudaticae* N31, by submerged fermentation (Silva et al. 2014). The milk clothing activity of the enzyme was tested, and a value of 60.5 U/mL was reported. The enzyme showed high heat‐stability and better performance under slightly acidic conditions, suggesting that could be considered for dairy production (Silva et al. 2014). A recent study investigated the proteases produced by thermophilic *Anoxybacillus caldiproteolyticus* 1A02591 strain. When cultured with casein, different proteases were secreted, mainly metalloproteases and serine proteases, which displayed an optimum activity at 70°C and were able to degrade different proteins (Cheng et al. 2021). Further research could evaluate the potential of these proteases in the food industry.

#### 5.2.3 Laccases

Laccases are multi‐copper oxidoreductase enzymes with a great variety of substrates, such as phenolic compounds, aromatic amines, aniline compounds, and recalcitrant pollutants (Sharma, Ayothiraman, and Dhakshinamoorthy 2019). In the food industry, these enzymes could be used to improve or change the color of food and beverages. Laccase enzymes are used as an alternative to physical–chemical adsorbents in the wine industry, to avoid unwanted changes in the organoleptic characteristics (Minussi, Pastore, and Durán 2002) and also in the beer industry, to eliminate any excess oxygen from the final product, extending the beer's shelf life (Mate and Alcalde 2017). To our knowledge, few studies have explored laccases from thermophilic organisms. A study that employed a thermophilic *Bacillus* sp. PC‐3 isolate to produce extracellular laccase, showed that its maximum activity is at 60°C and pH 7 (Sharma, Ayothiraman, and Dhakshinamoorthy 2019). The results showed that the laccase used different substrates, including guaiacol, DMP, ABTS, and tannic acid. Furthermore, this enzyme was employed in functionalization of a chitosan film, improving antioxidant and antimicrobial properties (Sharma, Ayothiraman, and Dhakshinamoorthy 2019). Thus, this laccase could be considered for new applications in food‐related industries, such as packaging.

## 6 Acidophilic Microorganisms and Their Enzymes

Acidophiles are microorganisms whose growth can only occur optimally at pH < 3. They must be able to produce ATP while maintaining a gradient of many pH units across the cellular membrane in order to thrive at a low pH (Baker‐Austin and Dopson 2007). Acid‐stable enzymes like propionyl‐CoA synthase, two acetyl‐CoA synthetases, and lactate‐2‐monooxygenase that convert lactate to pyruvate are proteins present in some acidophilic organisms (e.g., *Ferroplasma acidiphilum*) which possess voluminous complementary ferrous proteins that underpin them at very low pH (Baker‐Austin and Dopson 2007). Since most acidophiles grow at extreme pH ranges, their acid‐stable enzymes could be applied in the production of catalysts and lubricants (Van den Burg 2003), and they could also be of potential use in food products that require the degradation of organic acids. Indeed, acidophiles have been mostly used for biomining of gold, iron, and copper, among other metals. However, they are also famously explored due to their acid‐stable EXZs for applications in microbial fuel cells, biomaterials production, pharmaceutical industry, animal feed, and food processing (Sharma et al. 2012; Ni et al. 2016; Quehenberger et al. 2017; Roberto and Schippers 2022; Vera et al. 2022).

### 6.1 Acclimation Mechanism of Acidophilic Microorganisms

The acidophiles use several mechanisms to acclimate and function. For instance, they restrict and purge protons and their effects from the cytoplasmic membrane in order to establish homeostasis, thus making it possible to attain circumneutral cytoplasmic pH similar to neutrophiles (Dopson et al. 2023). They also evolve through gene acquisition and transfer to cement their first and second layers of defense, exemplified by the *Fervidacidithiobacillus*, *Igneacidithiobacillus*, *Ambacidithiobacillus*, and *Acidithiobacillus* (González‐Rosales et al. 2022), and *Acidihalobacter* (Boase et al. 2022) genera. They respond to damage or harsh conditions through DNA repair or modification, and occasionally streamline their genome to survive and achieve growth in the acidic pH environment (Cortez et al. 2022). Some acidophiles like *Fervidacidithiobacillus caldus* use their FUR (ferric uptake regulator) to resist both acid and shock using their inherent regulatory genes that are of more or less equal crucial importance in several metabolic and cellular processes like biofilm formation and iron transport (Chen et al. 2020). Furthermore, these acidophilic organisms have strategically generated electrochemical barriers to protons in the form of ions (e.g., sodium and potassium) (Neira, Vergara, and Holmes 2022). They possess rigid impermeable membrane which resist protons influx and permeability. They also have first and secondary layers of antiproton pumps that readily participate in the removal of excess protons from the cytoplasm, while maintaining cytoplasmic buffering to combat acidification (Vergara et al. 2020).

### 6.2 Food Applications of Acidophilic Extremozymes

Acidophilic EXZs play significant roles in the food industry. They can be exploited based on their activity at low pH in fruit juice production and other food processes that make use of low pH. Their adaptability under severe conditions makes them usable in these food processing industries. The following are some EXZs derived from acidophilic microorganisms along with their promising or present applications in the food industry.

#### 6.2.1 Amylolytic Enzymes

The amylolytic enzymes are starch‐degrading enzymes which are largely grouped into α‐amylases, glucoamylases, and other amylases such as trehalosyl dextrin‐forming enzyme (TDFE). Amylases play crucial roles in starch conversion which is very critical to the textile, bread, and sugar industries. For instance, α‐amylase is used in the conversion or hydrolysis of raw starch from corn, cassava, and wheat, to maltodextrins. These are then subjected to the saccharification process involving glucoamylase and glucose isomerase to convert them into fructose syrups. The acidophilic organism to be first studied in detail for amylases was *Alicyclobacillus acidocaldarius*, and its α‐amylase is optimally active at pH = 3.

##### 6.2.1.1 α‐Amylases

Most α‐amylases are employed in the starch industry. The raw starch has a pH range of 3.2 to 3.6 but α‐amylases used for starch production need about pH = 6.5 to be optimally stable and active, thus necessitating the use of Ca^2+^. The pH is later adjusted in order to remove the ionic calcium, which inadvertently increases production costs. Therefore, acidophilic α‐amylase becomes very important in this regard because it is acidic, active, and stable optimally at pH = 3. This makes it to easily hydrolyze starch randomly at 75°C and 3.0 pH thereabout to produce the major end products which are maltose and maltotriose (Sharma, Kawarabayasi, and Satyanarayana 2012). Among the acidophilic microorganisms that have been reported to produce this acid‐stable amylase is *Bacillus* sp. YX‐1 which grows at pH = 4.5. Furthermore, it is easier for *B*. *amyloliquefaciens* amylase at pH = 3.0 to unfold from its molten globule‐like state and become usable in the production of starch and other foods (Asghari et al. 2004; Liu and Xu 2008; Sharma, Kawarabayasi, and Satyanarayana 2012).

##### 6.2.1.2 α‐Glucosidases

Lipid glycosylation, energy conversion, and the metabolism of carbohydrates are some main processes in which α‐glucosidases play key roles (Batrakov et al. 2002). Some recombinant α‐glucosidase clones like GlyFa1, GlyFa2, and α‐GluFa were produced *F*. *acidiphilum* genome. The enzymes were stable and could optimally perform at pH = 1.7–4.0 when intracellularly expressed in *E*. *coli*, suggestive of their usability in the aforementioned processes that are crucially needed in some food industries.

##### 6.2.1.3 Glucoamylases

Archaeal‐derived glucoamylases operate best at pH = 2.0 and 90°C, and have been reported from *Picrophilus oshimae*, *Thermoplasma acidophilum*, and *P*. *torridus*, among others which are known to only use starch as their main carbon source with the extracellular production of glucoamylase. Therefore, they exhibit more activity and stability at higher temperatures when compared to the bacterial and fungal glucoamylases (Serour and Antranikian 2002). These EXZs also show high specificity to more complex polysaccharides like starch than simple sugars, especially in comparison with other recombinant glucoamylases such as those obtained from *Thermoactinomyces vulgaris* and *S*. *solfataricus*. Recombinant forms come to the picture because thermoacidophilic organisms are difficult to cultivate with very low yields of enzymes. The first success was recorded when glucoamylase clone from *S*. *solfataricus* was conducted in a mesophilic host (Kim et al. 1995). This EXZ could act on maltotriose to liberate b‐d‐glucose obligately and is thus utilized in low‐calorie beer brewing, baking, alcoholic hydrolysis of whole grain, and fructose and dextrose syrups production.

##### 6.2.1.4 Trehalosyl Dextrin‐Forming Enzyme (TDFE)

TDFE could hydrolyze trehalose, which is a non‐reducing sugar with α‐1‐1‐linked molecules of glucose. Trehalosyl dextrin is reactively produced from dextrins by TDFE via the conversion of α‐1, 4‐glucosidic linkage to an α‐1, 1‐glucosidic linkage and trehalose‐forming enzymes (TFEs). The TFEs are maltooligosyltrehalose trehalohydrolase and maltooligosyltrehalose synthase and they cleave the α‐1, 4‐glucosidic linkage to α‐1, 1‐glucosidic linkage trehalosyl dextrins. The reaction series lead to the formation of trehalose from starch. The uses of trehalose range from food stabilizer, sweetener, and preservative, to medicinal and cosmeceutical applications (Richards et al. 2002).

#### 6.2.2 Proteases

Collagens are proteins quite important in medicine and the functional food industries. Acidophilic bacteria like *Bacillus* strain NTAP‐1 are reportedly producers of heat‐stable collagenase. This EXZ has an optimum pH = 3.9 and has been used to hydrolyze Azocoll with partial EDTA inhibition (Nakayama et al. 2000). Most acidic proteases are used in the production of cheese, a major example is renin.

#### 6.2.3 Xylanases

Xylanases which are acid‐stable have been produced from acidophilic microorganisms like *A*. *capsulatum*. *A*. *capsulatum*'s Xyn A gene possesses Glu 167 and Glu 282 conserved glutamates which hydrolyze *p*‐nitrophenyl‐b‐d‐cellobioside, carboxymethylcellulose, and xylan (Inagaki et al. 1998). Xylanases are also produced from *S*. *solfataricus* strain Oα with reported stability and activity on carboxymethylcellulose at pH = 3.5 and 95°C. When combined with SSO1354 strain to act upon xylan and cellulose, xylose and glucose were produced, respectively, indicative of the role of xylanases in the saccharification process (Maurelli et al. 2008).

## 7 Alkaliphilic Microorganisms and Their Enzymes

Alkaliphilic microbes are those found in alkaline environments at various econiches of the earth. Some human activities or geological processes could have created these alkaline conditions and environments, but a greater contributive factor could be ascribed to the activities of neutralophilic microbes exhibiting certain reactions. As a group of extremophiles, alkaliphilic bacteria are quite important and crucial to the food industry because novel microorganisms are found and cultivated from their natural habitats from natural habitats along with the production of their enzymes.

### 7.1 Acclimation Mechanism of Alkaliphilic Microorganisms

Alkaliphiles have adapted through the maintenance of intracellular pH homeostasis by keeping lower cytoplasmic pH against external environments, protecting their cell envelope, alkalinizing their own cells, bio‐energization, adapting to low nutrient bioavailability, and producing optimally stable extracellular biomolecules (Yumoto 2002; Fujisawa et al. 2010; Krulwich et al. 2011; Yokaryo and Tokiwa 2014; Saier Jr et al. 2016; Mamo 2019). The microorganisms mainly maintain pH homeostasis via H^+^ acquisition from the extracellular environment, blockage of OH^−^ from the extracellular environment, cytoplasmic H^+^ leakage reduction, and organic acid production. The cell envelope is key to the survival of the cell due to its ability to maintain cellular contents and allow material exchange between the cell and its environment in a controlled manner. For instance, Gram‐positive bacterial cells like those from *Bacillus* have different types of polysaccharides such as teichuronic and teichoic acids, which have been reported to crucially participate in cell wall function, a part of the cell envelope (Mamo 2019). Of equal importance are the roles of cell membrane lipids, lipopolysaccharides and S‐layer proteins (e.g., SlpA from *B*. *pseudofirmus* OF4) (Gilmour et al. 2000; Ito and Aono 2002; Silipo et al. 2004; Corsaro et al. 2006; Fujinami and Ito 2018) which aid the acclimation of the alkaliphiles even in severe climes.

Bio‐energization is carried out with the aid of ATP synthase which is embedded in thylakoids (in the case of photosynthetic cyano‐alkaliphiles), rendering the organism resistant to extracellular low H^+^ concentrations (Nevo et al. 2007; Schneider et al. 2007). The cytochrome plays a crucial role in the respiratory system of alkaliphiles along with some enzymes that aid cellular energy transfer. The increase in cytochrome content has been linked to pH increase; while studies on soluble cytochrome c‐552 of the alkaliphilic *Pseudomonas alcaliphila* showed that it can highly retain and reserve electron in periplasm within the alkaline pH range (Matsuno, Yoshimune, and Yumoto 2011; Matsuno and Yumoto 2015). Alkaliphiles also alter biomolecules production or expression via switching to alkali‐tolerant variants, producing more pH‐stable biomolecules, generating pH‐labile biomolecules, and activating protein damage repair (Verdolino et al. 2008; Hong et al. 2012; Dahl et al. 2015).

### 7.2 Food Applications of Alkaliphilic Extremozymes

Alkaliphilic microorganisms produce EXZs that are optimally active at pH > 9. The enzymes are highly stable under alkaline conditions due to their low amount of glutamate residues and high amount of arginine and histidine residues (Fujinami and Fujisawa 2010). Other than the detergent, bleaching, and leather industries where alkaliphilic enzymes are utilized for example, to improve the finished product color and appearance, they have also been used in the food industry.

#### 7.2.1 α‐Amylases

These alkaliphilic enzymes are optimally active at the pH of 10. Although they can undergo inactivation when exposed to 8 M urea, they are not affected by EDTA (up to 10 mM concentration). Starch is hydrolyzed by alkaliphilic α‐amylases into maltotriose, maltose, and glucose. The alkaliphilic *Bacillus* strain GM8901 was reported to yield 5 maltotetraose‐forming alkaline amylases in a culture broth (Kim et al. 1995), while *Bacillus alcalophilus* subsp. *halodurans* subsp. nov. reportedly produced an alkaline amylase in its strain NRRL B‐3881, which was also reported (Boyer, Ingle, and Mercer 1973). The alkaline amylases all have their optimal activities at the pH = 9–10 and possess starch degrading ability. The *Bacillus* sp. strain IMD370 alkaline amylase also hydrolyzed cornstarch to glucose, maltose, maltotriose, and maltotetraose at pH 8 (Kelly et al. 1995). Therefore, these EXZs have a wide variety of applications in the starch, baking, and brewing industries.

#### 7.2.2 Cyclomaltodextrin Glucanotransferases (CGTases)

These group of enzymes are also produced by the alkaliphiles. So many of the EXZs have been isolated from different bacterial strains. An example is a thermostable CGTase obtained from *Bacillus stearothermophilus* ET1 which has optimal activity at pH = 6 and 80°C (Chung et al. 1998). The enzyme could liquefy and convert 13% w/v cornstarch solution to cyclodextrin at the rate of 44%. The researchers also recorded the production of α‐, β‐, and γ‐cyclodextrins in the reaction. Cyclodextrins are important in the separation of enantiomers and isomers, catalysis of reactions, and detoxification of waste products. The use of CGTase becomes necessary to increase the production of cyclodextrins from starch and to reduce or avoid toxic and organic wastes along with the added value of economical cost of production (Horikoshi 1999). This is very critical in the corn starch production industry.

#### 7.2.3 Alkaline Lipases

The alkaliphilic enzymes were usually known for their applications in the soap and detergents industries but some anomalies do occur to extend their applications beyond these. The thermophilic alkaline lipase‐producer *Bacillus* sp. was isolated from Yellowstone National Park hot‐spring area (Wang et al. 1995). It is capable of producing lipases which have optimal activity at pH = 9.5 and 60°C. It acted well on natural fats, oils, and triglycerides with fatty acids, indicative of its paramount application in the food industry, especially emulsion‐based products.

#### 7.2.4 Pectinases

Pectin are soluble polysaccharides present in fruits and are used as thickeners in the baking and pharmaceutical industries. Pectin can bind to ingested foods intestinally while adding bulk to stool thereby aiding stool formation and may help in reducing the amount of cholesterol absorbed from foods. Pectinases therefore are of importance in the hydrolysis of pectin. They have an optimum activity pH of 10. The alkaliphilic bacterium *Bacillus* sp. strain GIR 277 was reported to produce pectic lyase with optimal activity pH = 9.5 (Yoshihara and Kobayashi 1982). In a later study, pectinase and xylanase were produced from alkaliphilic bacteria designated as NT‐2, NT‐6, NT‐33, and NT‐82, of which NT‐33 showed a significant degumming capacity on ramie (Cao, Zheng, and Chen 1992).

## 8 Halophilic Microorganisms and Their Enzymes

Halophilic bacteria have the ability to thrive in environments highly rich in salts. They are extremophiles within the Archaea, Bacteria, and Eukarya groups having a metabolic diversity of the methanogens, fermenters, aerobic heterotrophs, oxygenic and anoxygenic phototrophs, denitrifiers, and sulfate reducers (Quillaguamán et al. 2010; Liu et al. 2019).

### 8.1 Acclimation Mechanism of Halophilic Microorganisms

They have acclimated well by accumulating and producing compatible solutes that stabilize and absorb both stress and shock in their cells. These compatibles are biostructures targeting hypersaline, thermophilic, and psychrophilic adaptative conditions (Mokashe, Chaudhari, and Patil 2018). They have also adapted by maintaining homeostasis via the control of salt levels using K^+^ ions influx to balance osmotic pressure in the cellular cytoplasm while the Na^+^ ions are released simultaneously (Williams 2020). This mechanistic process is often utilized by multifarious halophiles to attain osmoregulation and adapt their intracellular proteins to hypersaline conditions, increasing the cytoplasmic salt levels to that of the surrounding environment.

### 8.2 Food Applications of Halophilic Extremozymes

The ability of halophiles to use saline water, grow at high pH, use mixed substrates, and produce negatively charged EXZs is valuable for the food industry. The halophilic microbes can secrete extracellular EXZs which include amylases, lipases, proteases, xylanases, and cellulases, capable of hydrolyzing reactions in hypersaline concentrations (Liu et al. 2019).

#### 8.2.1 Halophilic α‐Amylases

These EXZs catalyze the hydrolysis of starch's α‐1, 4‐glycosidic linkages and are widely applicable in several industries including foods. Halophilic amylases have not been widely studied but they potentiate better utility and performance based on their acclimation to being active and stable in saline environments or samples. They also feature lower water activity, alkaline pH stability, and high‐temperature ranges. Therefore, they play crucial roles in the saccharification processes of starch in order to produce maltooligosaccharides which are essential components of various food products. They also produce maltotetraose, maltose, maltotriose, and various types of glucose‐unit molecules, all of which are very important in the food production processes (Mesbah and Wiegel 2014; Kumar and Khare 2015; Kumar et al. 2016).

#### 8.2.2 Proteases

Proteases from halophilic microorganisms like *Bacillus* sp., *Pseudoaltermonas* sp., *Salinivobrio* sp., *Salicola* sp., *Halobacillus* spp., *Filobacillus* sp., *Chromohalobacter* sp., *Nesterenkonia* sp., and *Virgibacillus* sp. have been isolated and characterized (Hiraga et al. 2005; Amoozegar et al. 2007; Karbalaei‐Heidari, Amoozegar, and Ziaee 2009; Moreno et al. 2009; Shivanand and Jayaraman 2009; Vidyasagar et al. 2009). The EXZs have optimal activity and stability within the broad pH range of 5–10, and are useful in the food production processes involving saline conditions, especially in the processing of fish, meat, soy sauce, and other protein‐rich fermented foods (Setyorini et al. 2006; Setati 2010).

#### 8.2.3 Xylanases

Xylanases are known for their key role playing in the hydrolysis of xylan and are thus used in the baking industry to improve dough's organoleptic attributes. A few of halophilic xylanases have been studied including those from marine organisms and hypersaline bacteria like *Glaciecola mesophila*, *Chromohalobacter* sp., and *Nesterenkonia* sp. (Govender, Naidoo, and Setati 2009; Guo et al. 2009; Prakash et al. 2009). They generally show activity and stability within the pH = 6–11 and at temperatures > 60°C. These EXZs are increasingly popular for their prospective uses in bioethanol production as cellulosic materials have to be subjected to pretreatment and hydrolysis into the desirable and fermentable sugars (Wang et al. 2009).

## 9 Conclusion

With rising environmental and industrial issues, interest in biocatalytic conversion sources has recently increased. Enzymes are found in a wide range of food products. The focus of the study has moved from the terrestrial to the extreme climate in recent years. Some novel studies have focused mostly on enzymes that work under acute conditions. Therefore, extremophilic bacteria are a valuable source of new enzymes. High temperatures, acidic pH, high salinity, high radiation, low water activity, and high metal concentrations are only a few of the environmental variables for which these microbes have developed specific routes and molecular mechanisms. Due to their capacity to catalyze biochemical processes in extreme circumstances, EXZs are a potential family of enzymes whose characteristics may be investigated, leading to prospective exploitation in the food sector.

## Author Contributions

**Tolulope Joshua Ashaolu:** visualization (lead), writing – original draft (equal), writing – review and editing (lead). **Tanu Malik:** conceptualization (lead), writing – original draft (equal). **Rakhi Soni:** writing – original draft (equal). **Miguel A. Prieto:** writing – original draft (equal). **Seid Mahdi Jafari:** supervision (lead), visualization (supporting), writing – review and editing (supporting).

## Ethics Statement

The authors have nothing to report.

## Conflicts of Interest

The authors declare no conflicts of interest.

## Data Availability

The research findings of this study are available upon request from the corresponding author.
